# Experience with dalbavancin use in various gram-positive infections within Aberdeen Royal Infirmary OPAT service

**DOI:** 10.1007/s15010-023-02152-2

**Published:** 2024-01-02

**Authors:** James C McSorley, Darshini Reyes, Ivan Tonna, Vhairi Bateman

**Affiliations:** 1 School of Medicine, Medical Sciences and Nutrition, University of Aberdeen, Aberdeen, UK; 2 Department of Pharmacy, NHS Grampian, Aberdeen Royal Infirmary, Aberdeen, UK; 3 Infection Unit, NHS Grampian, Aberdeen Royal Infirmary, Aberdeen, UK

**Keywords:** Dalbavancin, Lipoglycopeptides, Osteomyelitis, Bacteraemia, Cellulitis, OPAT

## Abstract

**Purpose:**

Dalbavancin, approved in 2014 for Gram-positive acute bacterial skin and skin structure infections (ABSSSI), has pharmacokinetics enabling treatment with one or two doses. Dalbavancin might be useful in outpatient parenteral antibiotic therapy (OPAT) of deep-seated infections, otherwise requiring inpatient admission. We documented our experience with pragmatic dalbavancin use to assess its effectiveness for varied indications, on- and off-label, as primary or sequential consolidation therapy.

**Methods:**

Patients prescribed dalbavancin between 1 December 2021 and 1 October 2022 were screened for demographics of age, sex, Charlson comorbidity index (CCI), allergies, pathogens, doses of dalbavancin, other antibiotics administered and surgery. Where available, infection markers were recorded. The primary outcome was a cure at the end of treatment. Secondary outcomes included any adverse events and for those with treatment failures, response to salvage antibiotics.

**Results:**

Sixty-seven per cent of patients were cured. Cure rates by indication were 93% for ABSSSI, 100% for bacteraemia, 90% for acute osteomyelitis, 0% for chronic osteomyelitis, 75% for native joint septic arthritis and 33% for prosthetic joint infection. Most bone and joint infections that were not cured did not have source control, and the goal of treatment was suppressive. Successful suppression rates were greater at 48% for chronic osteomyelitis and 66% for prosthetic joint infections. Adverse events occurred in 14 of 102 patients.

**Conclusion:**

This report adds to clinical experience with dalbavancin for off-label indications whilst further validating its role in ABSSSI. Dalbavancin as primary therapy in deep-seated infections merits investigation in formal clinical trials.

## Introduction

Management of deep-seated Gram-positive infections poses a challenge for clinicians. Amongst such entities are osteomyelitis, endocarditis, deep abscesses, pyomyositis, bacteraemia and septic arthritis [1, 2]. Several factors conspire to make these conditions difficult to treat. The presence of devitalised tissue such as bony sequestrum, necrotic debris and pus provides an immune-privileged nidus of infection [3]. The avascularity, lowered pH and redox potential of these infective loci oftentimes impede access and bactericidal activity of extraneous antibiotics in addition to components of the immune system [3, 4]. Foreign bodies, including indwelling prostheses, are also frequently involved in deep-seated infections, for the same reasons [5, 6]. These infections typically involve a large inoculum of organisms many of which may be metabolically dormant and/or incorporated within protective biofilm, rendering them less vulnerable to destruction by antibiotics and the host immune response [3, 4]. Where surgery or other mechanical means of source control are not feasible, antibiotic therapy is not typically curative and the goal of management is merely suppressive, aiming to improve quality of life for the patient [7].

Although there is arguably a wider choice of agents available to treat Gram-positive infections than their Gram-negative counterparts, the available options are still not ideal [8, 9]. Few oral agents offer sufficient bioavailability to be utilised in these infections and those that do present substantial limitations. Linezolid, fusidic acid and rifampicin have each been associated with haematological dyscrasias and are all potentially hepatotoxic, particularly with prolonged use, the latter also being unsuitable for monotherapy owing to the potential for rapid selection of mutational resistance during therapy [10–14]. Rifampicin also acts as a potent inducer of hepatic cytochrome P450 enzymes, reducing the effects of partnered drugs which are substrates of these, such as fusidic acid, resulting in de facto monotherapy and potentially selecting for rifampicin resistance when such combinations are utilised [15]. Pristinamycin has been associated with occasional reports of fatal toxic epidermal necrolysis [16]. The use of clindamycin and fluoroquinolones is jeopardised by a relatively high background rate of resistance amongst staphylococci and the attendant risk of *Clostridioides difficile* infection [17, 18]. Fluoroquinolones are approved only for use in adults, and their use also entails the risk of rare yet serious adverse events including QT interval prolongation, ruptured aortic aneurysm, tendonitis and retinal detachment [19]. Co-trimoxazole, doxycycline and minocycline are not ideal from an antimicrobial stewardship perspective given that they each exert unnecessary selective pressure for resistance in Gram-negative organisms, as do fluoroquinolones [20–22]. Many of these oral options, though generally active against staphylococci, are not consistently active against streptococci and other organisms that may be involved in complex infections [23]. In any case, there is always a risk of non-concordance with oral therapy, especially amongst patients with complicating psychosocial factors [24]. As a result, intravenous agents are typically advised in deep-seated infections, at least in the initial intensive phase of therapy, commonly employed agents include β-lactams, vancomycin, teicoplanin and daptomycin [25]. Such intravenous therapy poses several logistical challenges including inpatient admission or the need for regular attendance at OPAT clinics, central line insertion and oftentimes therapeutic drug monitoring [8, 25]. This is costly and must be undertaken by skilled personnel. Moreover, the invasive nature of central line placement can leave patients vulnerable to further iatrogenic harms including thrombosis and Gram-negative line-related infections [8, 25].

Dalbavancin, a lipoglycopeptide antibiotic for intravenous administration, has the potential to obviate most of these problems. It is the dimethylaminopropyl amide derivative of the naturally occurring compound A40926; itself a secondary metabolite of an actinomycete, *Nonomuraea gerenzanensis* ATCC 39727, isolated from an Indian soil sample in the 1980s [26]. Dalbavancin has an in vitro breath of spectrum qualitatively like that of the conventional glycopeptide antibiotics vancomycin and teicoplanin though with quantitatively greater potency [27]. Although all share a common mechanism of action, disrupting cell wall biosynthesis by binding to the terminal d-alanyl-d-alanine residues on murein precursor chains, modal MICs for *Staphylococcus* and *Streptococcus* spp. are 0.03–0.06 µg/ml, eightfold to 32-fold lower than those of vancomycin and teicoplanin (0.125–0.5 µg/ml) [28, 29]. The increased potency of dalbavancin owes to additional physicochemical mechanisms, such as the ability to dimerise and hydrophobically anchor into bacterial cytoplasmic membranes via its lipid side chains [27–29]. Animal studies have indicated that AUC/MIC is the parameter which most closely dictates the bactericidal action of dalbavancin and that it exhibits, to a degree, concentration-dependent kill kinetics [29]. This contrasts with vancomycin which displays time-dependent kill kinetics, despite sharing a common target [29]. In the case of *Staphylococcus aureus,* optimal AUC_24 h_/MIC ratios for dalbavancin are predicted to be 100–300, much lower than the 400–600 target suggested for vancomycin [30, 31]. Some in vitro data suggest that dalbavancin might possess other advantages over classical glycopeptides including greater potency against organisms in the biofilm state and the ability to suppress bacterial exotoxin production, though this has not been corroborated in clinical studies [32, 33]. Dalbavancin is highly active against most clinically relevant Gram-positive organisms with the notable exception of vancomycin-resistant enterococci or more rarely vancomycin-resistant *Staphylococcus aureus* (VRSA) carrying the *vanA* gene, as well as some species seldom encountered as pathogens such as *Enterocloster clostridioformis* [26–28]. Vancomycin-intermediate *Staphylococcus aureus* (VISA) strains typically have elevated minimum inhibitory concentrations (MICs) for dalbavancin though these still tend to be well below the susceptibility breakpoint [34]. Similar to the lipopeptide antibiotic daptomycin, dalbavancin has been found to be strongly synergistic with β-lactams in vitro through a phenomenon of collateral sensitivity known as the ‘see-saw’ effect, whereby increasing MIC for one agent brings about a corresponding drop in the MIC for the other, though it remains unclear whether this can be capitalised upon for enhanced clinical effect [35].

Dalbavancin displays linear, dose-dependent pharmacokinetics with a prolonged serum half-life of around 346 h, thus maintaining therapeutic unbound concentrations in blood, bone and synovial fluid for at least 6 weeks following a single intravenous administration [36, 37]. Previously reported concentrations measured 14 days after a single 1 g infusion were 4.1, 15.9 and 13.8 µg/g in cortical bone, synovium and skin, respectively, as compared to a plasma concentration of 15.3 µg/ml [36, 38]. Concentrations in blister fluid approximated 30 µg/ml, 7 days after an infusion of 1 g in another study. Dalbavancin is highly (~ 93%) protein bound in vivo, principally to serum albumin [36–38]. It does not interfere with hepatic cytochrome P450 enzymes and therefore has a low potential for deleterious drug interactions [39, 40]. Unlike vancomycin, dalbavancin has not been associated with ototoxicity or nephrotoxicity and ‘red man’ syndrome appears to be rarely, if ever, associated with its use [39, 40]. Two infusions of 1.5 g administered one week apart should provide 6–8 weeks of antibiotic activity that is theoretically comparable to that afforded by daily dosing with vancomycin or teicoplanin [39]. Dalbavancin was approved in 2015 for ABSSSI, but its use in deep-seated infections remains off-label though clinical experience backing this usage is now mounting [41–49]. The present study retrospectively evaluates clinical outcomes for patients with various infection types (ABSSSI, osteomyelitis, vascular graft infection, bacteraemia, septic arthritis and prosthetic joint infections) receiving dalbavancin either as primary or consolidation therapy.

## Methods

This retrospective observational study was conducted at Aberdeen Royal Infirmary, a large NHS teaching hospital serving a population of ca. 600,000 across the North of Scotland. After Caldicott approval, records of all patients receiving at least one IV dose of dalbavancin between December 2021 and October 2022 were screened. Patient age, sex, Charlson comorbidity index (CCI), allergies, causal pathogens, doses of dalbavancin and other antibiotics administered, receipt of surgery or other source control modalities were each noted in an anonymised database. Where available, changes from baseline values in C reactive protein (CRP) trend, liver enzymes and estimated glomerular filtration rate (eGFR) were noted as were any adverse events emerging during treatment. The primary outcome assessed was clinical cure versus failure or improvement at the end of treatment (i.e. 6 weeks from the last dalbavancin dose) as judged by the treating consultants and ongoing need, if any, for further antibiotic treatment. Where cure was confirmed, no further follow-up was deemed necessary but otherwise was continued indefinitely until cure had been achieved or the patient expired. Secondary outcomes included treatment-emergent adverse events as well as use of the drug as primary versus sequential consolidation therapy. For treatment failures, response to subsequently administered antibiotics was assessed. Primary dalbavancin therapy was defined as receipt of no more than 48 h of intravenous therapy with another agent active against the known or suspected pathogen. In cases where co-infection with dalbavancin-insensitive organisms was confirmed or suspected, additional agents specifically targeting those organisms were permitted within this definition of primary therapy, e.g. aztreonam or temocillin, for Gram-negative coverage or metronidazole for anaerobes. Consolidation therapy was defined as initial receipt of at least 48 h of another IV agent active against the target pathogen, e.g. β-lactams, glycopeptides or daptomycin, before completing the course of therapy with dalbavancin once blood cultures, if positive at baseline, had cleared. Two standardised dosing schedules were utilised for dalbavancin administration. For deep-seated infections, 1.5 g was infused as a loading dose followed 7 days later by a further 1.5 g; this was expected to provide 6 weeks of antibiotic activity, though blood levels were not measured. If there was a need for ongoing therapy, for instance, in suppressive treatment of chronic infection, the regimen could be extended thereafter by giving further 1.5 g infusions at monthly intervals with ongoing clinical review or switching to an appropriate orally administered agent. In the case of ABSSSI, a loading dose of 1 g was infused initially, and if required, 0.5 g could be given 7 days later according to response. A single patient with renal failure was treated with a personalised dosing schedule comprising a 0.75 g loading dose followed by half that amount given after one week. Cure was defined as cessation of all clinical signs and symptoms of infection as judged by the treating clinicians, with no further antibiotic treatment being required. Improvement or successful suppression was considered to have been achieved if key signs and symptoms such as pyrexia, purulent discharge, subjective pain scores and limited range of motion had initially resolved or lessened, but further antibiotic treatment was thought necessary to prevent recurrence.

## Results

In total, 102 patients were included, 71 male and 31 female. Patient ages ranged from 31 to 97 years with a median of 63. Breakdown by indication was as follows: 45 cases of ABSSSI, 35 cases of osteomyelitis, four cases of native joint septic arthritis, six of prosthetic joint infection and 12 of bacteraemia (Table 1). In some instances of bacteraemia, other indications were also listed for the same patient owing to haematogenous dissemination of organisms; 69 of 102 patients were cured indicating a net cure rate of 66.66%. Of the remaining 34 patients, 17 were considered clear treatment failures (16.66%) and 18 were deemed improved (17.65%).

**Table 1:** Summary of treated cases

	Total	Failures	Cures	Improved	Primary dalbavancin
Age (range)	31–97				62/102
Sex (M/F)	(71/31)	(12/7)	(50/19)	(9/7)	
1. ABSSSI	45	3	42	0	34
2. Osteomyelitis	35	14	9	12	24
Source control	10	1	9	0	7
No source control	25	13	0	12	17
3. Septic arthritis	10	2	5	3	4
Native joint	4	0	3	1	1
Prosthetic joint	6	2	2	2	3
4. Bacteraemia	12	0	12	0	0
Endovascular graft	1	0	1	0	0
IV Drug abuse	4	0	4	0	0
Dental extraction	1	0	1	0	0
Pneumonia	1	0	1	0	0
Discitis	2	0	1	0	0
ABSSSI	2	0	2	0	0
Other	1	0	1	0	0

From the 102 included patients, 69 Gram-positive organisms were isolated (Table 2). Note that in many instances, including the vast mast majority of ABSSSI cases, bacteriologic aetiology was not laboratory confirmed. Cases from which no organism was cultured but which had a significantly elevated titre of anti-streptolysin O antibody on serologic testing were assumed to be due, at least in part, to β-haemolytic streptococci; hence, these are included in the figures for those organisms. From several cases, more than one organism was isolated. Some organisms isolated from polymicrobial infections were of doubtful clinical import but are nonetheless included for completeness*.

**Table 2:** Gram-positive isolates

Pathogen	Total (clinical failures)
*Staphylococcus aureus,* methicillin-susceptible (MSSA)	27 (7)
*Staphylococcus aureus,* methicillin-resistant (MRSA)	4 (1)
Coagulase-negative *Staphylococcus spp.*	9 (4)
β-haemolytic *Streptococcus spp.* Groups A, B, C, G	13 (4)
Viridans group *Streptococcus spp.*	2 (0)
*Streptococcus pneumoniae*	1 (0)
*Enterococcus faecalis* (vancomycin-susceptible)	4 (2)
*Enterococcus faecium* (vancomycin-susceptible)	1 (1)
*Corynebacterium tuberculostearicum*	1 (0)
*Corynebacterium afermentans*	1 (0)
*Lactobacillus spp.*	1 (0)
*Kocuria spp.*	1 (0)
*Bacillus licheniformis*	1 (0)
Niallia circulans*	1 (0)
Clostridium sporogenes*	1 (0)
*Parvimonas micra*	1 (1)
Total	69 (20)

Fourteen patients in the series were considered to have suffered an adverse event during dalbavancin therapy. These are summarised in Table 3. Note that although four instances of acute kidney injury (AKI) and two cases of *C. difficile* colitis are included; these were not thought to be due to dalbavancin (Table 3).

**Table 3:** Adverse events occurring during dalbavancin therapy

Adverse events	No. of cases
Elevated hepatic enzymes	4
Acute kidney injury	4^a^
Drug fever	2
Maculopapular rash	2
*Clostridioides difficile* colitis	2^a^

^a^NB—These cases were not thought to be directly attributable to dalbavancin; three of four AKI cases occurred on a background of pre-existing chronic kidney disease, and the remaining case was in receipt of multiple nephrotoxic drugs including furosemide, ramipril and naproxen. Prior administration of other antibiotics, ceftriaxone in one instance and a combination of clindamycin and ciprofloxacin in the other were thought likely to be responsible for the two *C. difficile* infections

 Nineteen of 102 (18.6%) patients in this series, with ages ranging from 31 to 82 years, 12 male and 7 female, were considered clear treatment failures (Table 4). Amongst these failures were three cases of ABSSSI, two of prosthetic joint infection and 14 of osteomyelitis. Factors contributing to treatment failure were lack of source control (*n* = 13), presence of undrained collections (*n* = 3), indwelling orthopaedic metal work (*n* = 3), immunosuppression (*n* = 3), death from other causes (*n* = 3), inability to tolerate dalbavancin (*n* = 1) and co-infection with dalbavancin-insensitive organisms (*n* = 9). Note that failure was multifactorial in most instances with considerable overlap. Given the extended half-life of dalbavancin, not all patients who discontinued therapy were counted amongst the treatment failures as several achieved cure or improvement despite having received only a single dose of the drug.

**Table 4:** Summary of treatment failures

Failure no	Demographics	CCI	Indication	Description of failure	Pathogen
1	Male, 59	8	Chronic pelvic osteomyelitis	Deceased. Succumbed to Gram-negative sepsis despite switching to carbapenem	*Staphylococcus epidermidis, Klebsiella pneumoniae, Parvimonas micra*
2	Female, 58	2	Chronic prosthetic knee infection	Required switch to ceftriaxone for PICC infection due to *Proteus mirabilis*, original infection improved	*Staphylococcus lugdunensis*, *Staphylococcus caprae*
3	Female, 58	1	Multiple abscesses in axillae and groin 2֯ to hidradenitis suppurativa	Immunosuppressed (on adalimumab), pathogens eradicated but no clinical response	*E. faecalis* (vancomycin-susceptible)
4	Male, 60	4	Infected diabetic foot ulcer	No response, improved after switch to piperacillin/tazobactam after breakthrough Gram-negative bacteraemia	Group B *Streptococcus* (GBS), MSSA, *Klebsiella oxytoca*, *Citrobacter koseri*
5	Male, 82	4	Discovertebral (C7–T1) osteomyelitis with epidural abscess	Indwelling metalwork in spine, monoclonal gammopathy. Developed fever and rigors thought to be due to dalbavancin, improved on switch to teicoplanin	MSSA
6	Male, 44	4	Chronic pelvic osteomyelitis	Deceased, paraplegic with hindquarter amputation, succumbed to an intercurrent bilateral pneumonia	MSSA, *Escherichia coli*
7	Male, 59	7	Paraspinal abscess, infected L3–S1 fusion metalwork	Deceased, no source control. succumbed to metastatic colorectal cancer	Nil detected
8	Male, 54	5	Chronic osteomyelitis of distal tibia, fibula, navicular & metatarsals	Secondary to diabetic ulcer and Charcot foot. MSSA eradicated but required amputation. Marked sequestrum	MSSA, *Pseudomonas aeruginosa*
9	Female, 65	3	Chronic metalwork infection in elbow	No source control	*S. epidermidis*
10	Female, 63	7	Osteomyelitis of great toe	Dalbavancin not tolerated—maculopapular rash. resolved on switch to PO doxycycline	MSSA
11	Male, 31	0	Chronic infection of elbow and forearm	No response to several antibiotics. Polymicrobial infection, several organisms isolated over time. Underlying pathology under investigation	*S. epidermidis, Streptococcus mitis/oralis, E. coli, K. pneumoniae, Haemophilus parainfluenzae, Stenotrophomonas maltophilia, Mycobacterium obuense*
12	Female, 56	2	Chronic pelvic osteomyelitis with discharging sinuses	No source control; indwelling Harrington rods. Temporary improvement but relapsed	MSSA, *E. faecalis*
13	Female, 67	6	Chronic pelvic osteomyelitis with collection	Collection remained undrained	GBS
14	Male, 74	6	L5–S1 discovertebral osteomyelitis	Immunosuppressed—on mitomycin for bladder carcinoma	Nil detected
15	Male, 63	5	Lower limb cellulitis 2֯ to diabetic foot ulcers	MSSA eradicated but no clinical improvement	MSSA, *E. coli*, *P. aeruginosa*
16	Male, 67	4	Osteomyelitis of great toe	Required amputation	Nil detected
17	Male, 46	1	Osteomyelitis 5th toe	Type 1 diabetes mellitus, human immunodeficiency virus (HIV) + ve, required concurrent co-amoxiclav	Nil detected
18	Female, 44	3	Pelvic osteomyelitis 2֯ to pressure ulcer	Paraplegic. Switched to piperacillin-tazobactam due to septic shock and responded	Group A *Streptococcus* (GAS), *P. aeruginosa*
19	Male, 70	4	Upper limb osteomyelitis	Infected metalwork in situ, received concurrent aztreonam for Gram-negative coverage	MRSA *E. coli*

## Discussion

This report adds to accumulating clinical experience with off-label dalbavancin usage in complex and deep-seated Gram-positive infections. The results obtained confirm that dalbavancin is a useful and well-tolerated agent in ABSSSI, enabling administration of effective OPAT regimens with a convenient schedule of only 1–2 doses without the need for PICC insertion and its attendant risks. Only 14 of 102 (14%) included patients suffered an adverse event on therapy. Though uncertain, this is likely an overestimate as it includes events that were thought not to be related to dalbavancin administration; 42 of 45 ABSSSI treated in this series were cured, indicating a success rate of 93%. Of the 45 patients treated with dalbavancin for ABSSSI, 34 (76%) received it as primary therapy. Most cases of ABSSSI treated were cellulitis or infective bursitis. No cases of necrotising fasciitis were treated. It may be of particular interest to evaluate clinical utility of dalbavancin for necrotising infections in future trials given that, in vitro, it was recently shown to attenuate production of various protein exotoxins including Panton–Valentine leucocidin (PVL), toxic shock syndrome toxin 1 (TSST-1) and α-haemolysin by *S. aureus*, unlike vancomycin or β-lactams [31]. This therapeutic property is shared by the bacteriostatic protein synthesis inhibitors clindamycin and linezolid, making them especially useful for invasive infections [32]. Whether dalbavancin could exert similar superior efficacy in such infections remains unknown. Notably, one patient in this series was cured when treated with dalbavancin as a single agent for olecranon bursitis that was later confirmed to be due to a PVL-producing MRSA strain. Two of the clinical failures in ABSSSI involved polymicrobial infection of diabetic foot ulcers and it is probable that Gram-negative organisms contributed at least partially to these failures as both cases subsequently resolved  after switching to piperacillin–tazobactam treatment. The remaining treatment failure amongst the ABSSSI patients was in a case of hidradenitis suppurativa who was receiving immunosuppressant therapy with adalimumab; although GBS and *E. faecalis* were both eradicated on dalbavancin therapy, substantial clinical improvement was not achieved until after a change in regimen to  intravenous piperacillin–tazobactam and oral doxycycline. For bacteraemia, though the number of cases treated was small at only 12, consolidation therapy with dalbavancin was uniformly successful when instituted after initial bacteriologic clearance was achieved, within 48–96 h in all cases. This may suggest that OPAT with dalbavancin has the potential to expedite discharge of bacteraemic patients who would otherwise require, if not ongoing hospitalisation, 6 or more weeks of IV therapy, necessitating clinic attendance on at least a thrice weekly basis. Interestingly, two cases of *S. aureus* bacteraemia with septic emboli, both in IV drug users, received less than 7 days of standard  intravenous therapy with flucloxacillin before discharge on dalbavancin. In these cases, dalbavancin was offered as a compromise before each patient insisted on self-discharge against medical advice. Therefore, dalbavancin merits further study as a standalone agent for primary therapy of bacteraemia, a question not addressed here or, to our knowledge, elsewhere in the published literature. Randomised controlled trials of primary dalbavancin versus standard therapy; isoxazolyl penicillins for MSSA bacteraemia or vancomycin for MRSA could therefore be of future interest. There is also a knowledge gap concerning the potential utility of dalbavancin in combination therapy with, for example, β-lactams and/or rifampicin. There are theoretical grounds to suspect that such combinations may hold promise given that combinations of dalbavancin with β-lactams can elicit the synergistic ‘see-saw’ effect whilst both dalbavancin and rifampicin display in vitro activity against biofilm [8, 31, 38]. Although not explicitly examined as an outcome here, we did not note any trends in this series to suggest that patients receiving dalbavancin as sequential therapy after a β-lactam had superior clinical outcomes. No patients in the series received adjunctive rifampicin, and hence, we could not delineate whether this would have improved outcomes. In osteomyelitis, dalbavancin was largely curative in the acute phase but not in chronic cases, a finding replicated in other studies published to date [41–49]. Nevertheless, chronic osteomyelitis, particularly when debridement had been possible, frequently improved on dalbavancin as judged subjectively by reduced pain, lessening of discharge and improved mobility as well as objectively, in some cases, by CRP trend and lack of radiologically visible disease progression. Of the 14 osteomyelitis cases deemed to be clear therapeutic failures, 13 had no source control and were receiving dalbavancin with the aim of suppression rather than cure. The efficacy of therapeutic agents in chronic osteomyelitis is notoriously difficult to evaluate owing to the lack of a universally agreed upon and objective metric by which to assess clinical response. For this reason, few clinical trials have sought to address this issue, especially amongst patients in whom source control has not been possible. In agreement with other studies, dalbavancin therapy was found to be successful in cases of acute septic arthritis affecting native joints, but surgical source control was imperative for cure to be achieved in prosthetic joint infection [40, 43–45]. In these respects, dalbavancin, though not curative, was not obviously inferior to other agents widely used in suppressive therapy of bone and joint infections.

Based upon drug acquisition tariffs alone, a 6-week course of dalbavancin therapy (two infusions of 1.5 g) costs £3352.20 [50]. The equivalent costs to the National Health Service for 6-week courses of IV flucloxacillin (2 g QDS), vancomycin (1 g BDS), teicoplanin (0.8 g OD) and daptomycin (0.7 g OD), respectively, are £1008.00, £925.00, £614.88 and £5040.00 [50]. This suggests that flucloxacillin, vancomycin and teicoplanin, though not daptomycin, are more cost-effective options than dalbavancin for IV therapy of deep-seated Gram-positive infection. Crucially, however, these sums do not incorporate additional costs such as inpatient admission, therapeutic drug-monitoring or central line placement. The cost of PICC insertion alone has been estimated at £1000.00 [51]. Considering these additional expenditures, dalbavancin administered through OPAT may be a pharmacoeconomically sound option, at least for a subset of patients. The convenience of dalbavancin through OPAT may be particularly suited to patients with psychosocial obstacles to compliance such as immobility, lack of transport, substance misuse, mental illness, homelessness, nomadic lifestyle or incarceration. One of the treatment failures in our series (No. 2) resulted from delayed removal of a PICC line, and resulting *P. mirabilis* infection, upon switch from vancomycin to dalbavancin. This emphasises the potential benefit of dalbavancin use as this is given through a cannula that is removed soon after administration of the drug. Similarly, one of the two patients in this series developing *C. difficile* colitis probably did so because they received a single dose of ceftriaxone to ‘bridge’ their transition from flucloxacillin to dalbavancin. It is likely this could have been avoided had the patient been switched directly to dalbavancin.

The uniquely long terminal half-life of dalbavancin has raised theoretical concerns that it may have an unusual propensity to select for resistance during the prolonged elimination phase at the end of therapy [52, 53]. Given that this was a retrospective observational study, emergence of resistance during or after dalbavancin therapy was not specifically monitored. Emergent cross-resistance to dalbavancin, vancomycin and daptomycin in *S. aureus* has recently been reported after dalbavancin therapy and could have especially grave implications given the critical importance of these drugs as ‘workhorse’ agents for the management of β-lactam-resistant Gram-positive infections [52, 53]. Therefore, vigilant surveillance for resistance would be prudent, should dalbavancin ever be used on a wider scale. This is especially true where dalbavancin is used for suppressive therapy of chronic deep-seated infections where there is a high inoculum of organisms and source control has not been possible.

The retrospective, observational nature of this study poses some other critical limitations. In cases receiving dalbavancin as consolidation therapy, the type and dosage of antibiotics given beforehand for initial therapy could not be standardised. Prior use of oral antimicrobials in primary care could not be accounted for in some cases. Likewise, no matched control arm was included for comparison and to have done so in retrospect would have risked bias. Given that all patients were treated within the past year, follow-up periods and opportunity to detect recurrence were limited. Aside from the bacteraemic cases, no attempt had been made to identify causative pathogens in most patients as this would have required invasive sampling methods. This meant that it was not possible to comprehensively stratify outcomes by pathogen and we cannot exclude the possibility that some treatment failures were unknowingly the result of infection with unidentified organisms not fully sensitive to dalbavancin. No attempts were made to quantify susceptibility of clinical isolates to dalbavancin, for instance, by E-test or broth dilution methods. The pragmatic inclusion of ‘real-life’ patients with complex multimorbidity and polypharmacy may have confounded interpretation of outcomes in several areas. In cases of treatment failure, it was not possible to measure separately the relative contribution of distinct factors which were frequently multiple.

## Data Availability

Raw data not accessible in public domain, in line with NHS Scotland Caldicott principles.
